# Basic and clinical immunology – 3032. 18FDG-PET/CT findings (SUV-MAX) are correlated with survival in stage-4 squamous cell carcinoma

**DOI:** 10.1186/1939-4551-6-S1-P207

**Published:** 2013-04-23

**Authors:** Arzu Didem Yalcin, Metin Erkilic, Bulent Kargi, Aysegul Kargi, Burhan Savas, Antonia Dimitrakopoulou-Strauss, Ludwig Strauss

**Affiliations:** 1 Internal Medicine, Allergy and Immunology, Education and Research Hospital, Turkey; 2 Department of Nuclear Medicine, Akdeniz University Hospital, Turkey; 3 Medical Park Hospital, Turkey; 4 Department of Internal Medicine, Antalya Education and Research Hospital, Antalya, Turkey; 5 Department of Internal Medicine, Oncology Unit, Akdeniz University Hospital, Antalya, Turkey; 6 Clinical Cooperation Unit Nuclear Medicine, German Cancer Research Center, Im Neuenheimer Feld 280, 69120, Heidelberg, Germany

## Background

Gene promoter hypermethylation is now regarded as a promising biomarker for the risk and progression of lung cancer. Vit-D is a steroid hormone that is now widely accepted that it exerts several extraskeletal actions, including anti-tumorigenic and immunomodulatory effects in vitro and in vivo. Its potential in cancer prevention and treatment is currently under detailed investigation.With the prospect of more effective therapeutic options for advanced stage disease, there are still insufficient current follow-up procedures for lung cancer. Therefore, in the present study, we investigated the concentrations of sCD200,sTRAIL,Vit-D,Hcy in the peripheral blood of metastatic non-smalcelllungcancer(MNSCLC) patients. As well as the corresponding F-18-Deoxyglucosepositronemissiontomography (PET/CT) results were evaluate

## Methods

Consecutive 22 adenocarcinoma and15 squamous cell carcinoma patients of MNSCLC referred to our institute were included in this study.The sCD200, sTRAIL, Hcy and Vit-D concentrations in the serum samples from the37 NSCLC patients were analyzed using a TRAIL/APO2L ELISA kit (Diaclone, France) and Roche kit for Vit-D and Hcy according to the manufacturer's instructions. The absorbance of each patient on a spectrophotometer using 450 nm and the concentration of sTRAIL(pg/mL), sCD200(pg/mL), Hcy(mmol/L) and Vit-D(ng/mL) was measured.

## Results

levels of sCD200, sTRAIL, Vit-D, Hcy are not to be corelated with survival in patients with MNSCLC.In patients with squamous cell carcinomaSUVMAX and survival are found to be corelated. The correlation is r=-0.6954, p=0.004, n=15.The data were evaluated using the StataMP software package, version 12.1 (StataCorp,College Station, TX,USA) on a Mac Pro 2*x*2.93 GHz, 2*6 Core Intel Xeon system with 24GB RAM using Mac OSX10.7.4(Apple, Cupertino,CA,USA).An error level of p<0.05 was generally used for the statistical analysis. The Wilcoxon matched pairs signed rank test was used to assess differences in the variables prior and three months after onset of treatment. Correlation tables were calculated for all variables. Furthermore, a multivariate correlation/regression analysis was applied to the data, using the survival as the dependent variable.

## Conclusion

F-18-Deoxyglucose(FDG), a glucose analogue, is an advanced imaging technique and allows a highly sensitive whole body search for malignant foci, which are detected by their increased glucose metabolism versus benign tissues, and successful scanning has been performed in a wide variety of cancers.

